# Achieving high strength and ductility in ODS-W alloy by employing oxide@W core-shell nanopowder as precursor

**DOI:** 10.1038/s41467-021-25283-2

**Published:** 2021-08-20

**Authors:** Zhi Dong, Zongqing Ma, Liming Yu, Yongchang Liu

**Affiliations:** State Key Laboratory of Hydraulic Engineering Simulation and Safety, School of Materials Science and Engineering, Tianjin University, Tianjin, China

**Keywords:** Mechanical properties, Metals and alloys

## Abstract

With excellent creep resistance, good high-temperature microstructural stability and good irradiation resistance, oxide dispersion strengthened (ODS) alloys are a class of important alloys that are promising for high-temperature applications. However, plagued by a nerve-wracking fact that the oxide particles tend to aggregate at grain boundary of metal matrix, their improvement effect on the mechanical properties of metal matrix tends to be limited. In this work, we employ a unique in-house synthesized oxide@W core-shell nanopowder as precursor to prepare W-based ODS alloy. After low-temperature sintering and high-energy-rate forging, high-density oxide nanoparticles are dispersed homogeneously within W grains in the prepared alloy, accompanying with the intergranular oxide particles completely disappearing. As a result, our prepared alloy achieves a great enhancement of strength and ductility at room temperature. Our strategy using core-shell powder as precursor to prepare high-performance ODS alloy has potential to be applied to other dispersion-strengthened alloy systems.

## Abstract (web summary)

Aggregation and coarsening of the second-phase oxide particles at grain boundaries have been a bottleneck for improving mechanical properties of oxide-dispersion-strengthened (ODS) alloys. Here the authors employ core-shell nanopowder precursors to achieve uniform dispersion of oxides in ODS alloys.

## Introduction

As for conventional engineering structural materials, improving their strength without sacrificing their ductility or enhancing their strength and ductility simultaneously has been a key goal for their various critical applications. For this purpose, numerous strategies have been taken, such as the formation of nanotwins in materials^1,2^, obtaining bimodal/multimodal grain structure^3,4^, and introduction of intragranular nano dispersoids^5,6^ or gradient structure^7^. Among them, the introduction of nano dispersoids has drawn considerable attention due to its universal applicability and has achieved success in many systems^8,9^. Once the second-phase nano dispersoids are introduced into the metal matrix, a large number of dislocations are expected to be pinned and accumulated within the matrix grain interior when they confront these dispersoids, giving rise to the enhanced strain hardening rate and ensuing high ductility^6,10^. Besides, the matrix can also be strengthened greatly by these dispersoids via an interaction between dislocations and nano dispersoids (either Orowan looping or particle shearing)^6,11^. Especially, the high-temperature microstructural stability benefiting from the introduction of second-phase dispersoids further highlights its application superiority^12^.

In recent decades, both the ex-situ and in-situ methods have been developed to homogeneously introduce the desirable second-phase dispersoids into different metal matrixes^13–15^. The nano dispersoids or nanoprecipitates introduced via the in-situ method are mainly synthesized through thermo-mechanical treatment or chemical reaction^15^. Thus, they generally exhibit good thermodynamic stability, fine size, and uniform distribution in metal matrix^13^. As a result, the in-situ dispersoid-strengthened engineering materials possess good mechanical properties and excellent high-temperature service capacity. Typical alloys strengthened by in-situ precipitations include Cu alloys^16^, Al alloys^17^, and stainless steels^18^, etc. Taking the Al-Sc binary alloy for example, the addition of Zr and Er elements can generally promote the formation of coarsening-resistant coherent L1_2_-Al_3_(Sc, Zr, Er) nanoprecipitates. Their ultrafine size (3–8 nm), consequently, results in a prominent strength enhancement from 243 MPa to 451 MPa^17,19^. More importantly, the coherent interfacial relationship between nanoprecipitates and matrix will not damage the ductility of materials while improving their strength. For instance, the precipitation of coherent B2 nanoparticles (3–5 nm) in a body-centered cubic (BCC) martensite matrix can render a Fe-17Ni-6.2Al-2.3Mo-0.48Nb-0.37C-0.05B steel with a high yield strength of 1.9 GPa and a total elongation of 8.2%^20^. Therefore, ultrafine and coherent nano dispersoid has become the new favorite of many alloy materials.

However, as for some alloy systems whose second-phase dispersoids can hardly be introduced by the in-situ method, the ex-situ method has to be generally adopted. Through powder metallurgy and various casting techniques^21,22^, nano-ceramic or intermetallic particles, such as oxides and carbides^23–25^, are introduced into the metal matrix, producing many materials with attractive physical and mechanical properties. However, with radically different physico-chemical properties from the matrix, these ex-situ nano-ceramic or intermetallic particles tend to aggregate and coalesce at the grain boundary of the metal matrix and form a semi-coherent or incoherent interface with matrix^23^, which largely weakens their strengthening effect compared with the in-situ coherent ultrafine nanoprecipitates mentioned above^14^. In addition, due to the deformation incompatibility, severe stress concentration could be easily induced at the semi-coherent or incoherent interfaces between these ex-situ second-phase particles and matrix, leading to crack formation and then the degradation of ductility of materials^6^. Therefore, in terms of the ex-situ second-phase particles strengthened alloy systems, how to introduce these particles through a method just like the in-situ approach above, i.e., full lattice coherency with matrix, ultrafine size, and completely intragranular distribution, has become the key to further develop high-performance second-phase particles strengthened alloys.

In this work, we successfully disperse ceramic oxide nanoparticles uniformly within the grains of metal matrix accompanying with the intergranular oxide particles completely disappearing, finally preparing high-performance oxide dispersion strengthened alloy. The key of our strategy is to employ the oxide@W core-shell structural composite nanopowders as precursor for the low-temperature sintering of desirable W-based ODS alloy. As a result, high-density oxide nanoparticles are introduced within the W grain interior well. These intragranular oxide nanoparticles with a size of only 1–3 nm present full lattice coherency with the surrounding W matrix. After high-energy-rate forging (HERF) processing, this alloy exhibits a large enhancement of both strength and ductility, better than previously reported pure W or second-phase particles strengthened W alloys.

## Results

### Mechanical properties

**Fig. 1:**
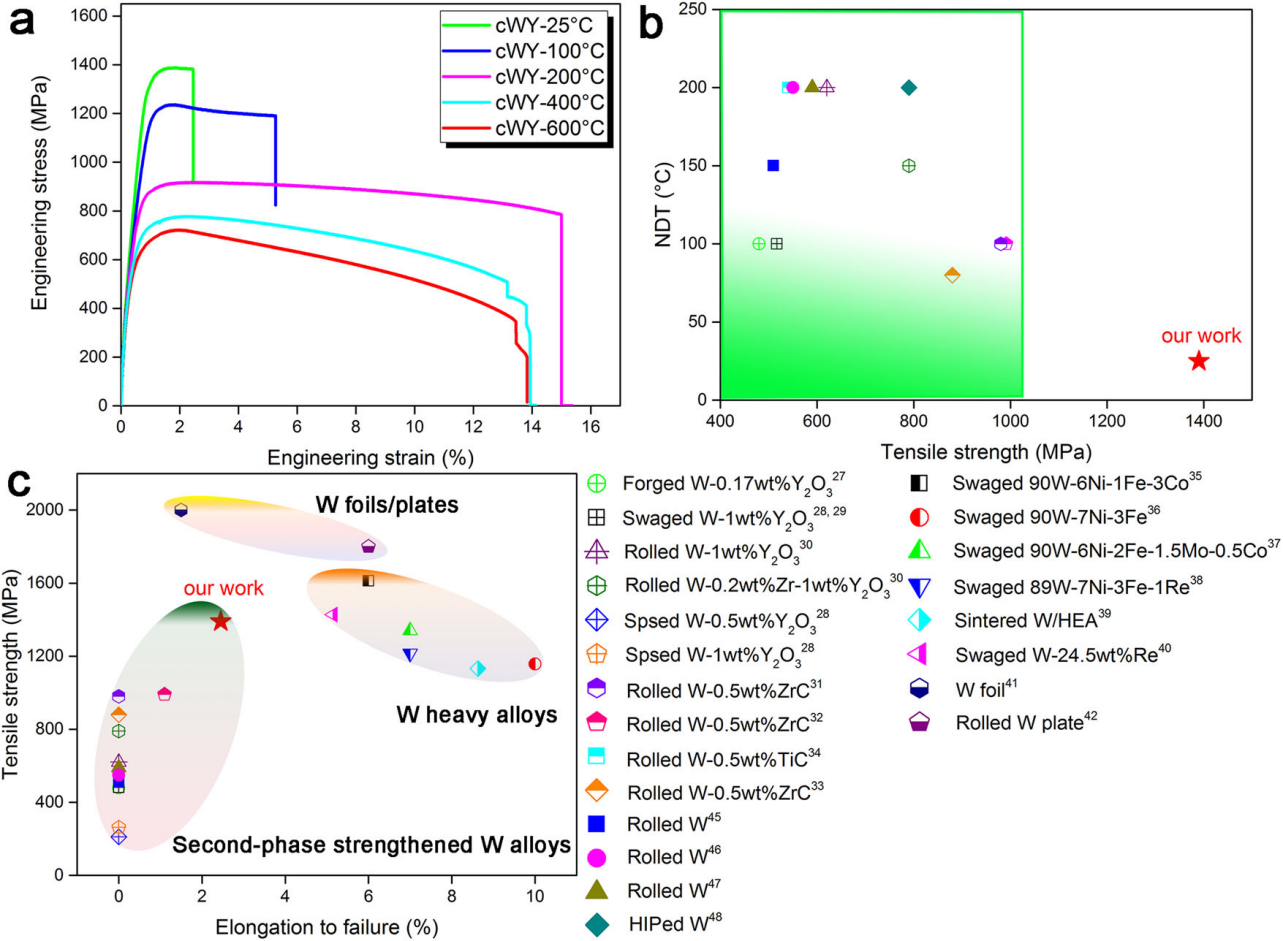
Mechanical properties of the cWY alloy. **a** Tensile curves, tested at temperatures from 25 to 600 °C. **b** Comparison of nil ductility temperature (NDT) between the cWY alloy and other established W-based alloy systems. **c** Comparison in strength and ductility for the cWY alloy prepared in this work, relative to several types of established W-based alloys and pure W. The reference systems include W-Y_2_O_3_ alloys^27–30^, W-ZrC alloys^31–33^, W-TiC alloy^34^, W heavy alloys^35–38^, solution strengthened W-based alloys^39, 40^, and highly deformed W plates/foils^41, 42^ and pure W^45–48^.

Fig. 1a shows the engineering stress-strain curves of our prepared cWY alloy (using the oxide@W core-shell powder with a nominal composition of W-0.5wt.%Y_2_O_3_ as precursor) at a temperature from 25 to 600 °C. Unlike traditional pure W or W-Y_2_O_3_ alloys, the cWY alloy presents obvious ductile fracture even at room temperature, with a uniform elongation of 1.05 ± 0.13% and total elongation (TE) of 2.50 ± 0.21%. This result indicates that the nil ductility temperature (NDT) of the developed alloy is below 25 °C, breaking the brittle feature of traditional W-based alloys at low temperature (see the NDT of various W-based alloys in Fig. 1b). For comparison, the tensile curves of W-Y_2_O_3_ alloy produced under the same process conditions but using traditional W-0.5wt.%Y_2_O_3_ composite nanopowder as precursor (denoted as WY alloy) are provided in Supplementary Fig. 1. Although it possesses higher strength at high temperatures due to the refined grains, the NDT of traditional WY alloy is between 200 °C and 400 °C, suggesting its brittle feature at low temperature. The maximum yield strength and ultimate tensile strength (UTS) of this cWY alloy at room temperature are 1210 ± 15 MPa and 1390 ± 18 MPa, respectively. When compared with the literature data of traditional second-phase dispersion strengthened W-based alloys (see Fig. 1c), it can be found that a remarkable strengthening effect occurs on this cWY alloy with simultaneous enhancement in ductility, which is the opposite of the strength-ductility trade-off relationship for traditional W-based alloys^26^. The cWY alloy exhibits about a 75.9% increase in UTS when compared with the optimal congeneric W-Y_2_O_3_ alloy (TE of 0%) reported in the literatures^27–30^ and 43.8% increase in UTS and 127.3% increase in TE when compared with the optimal W-ZrC alloy reported in the literatures^31–34^. Therefore, in second-phase dispersion strengthened W-based alloys, the developed cWY alloy exhibits a better combination of strength and ductility. To date, besides the cWY alloy in this work, the W-based alloys with appreciable low-temperature ductility (>3%) and high strength (>1 GPa) also include W heavy alloys^35–38^, solution strengthened W-based alloys^39,40^ and highly deformed W plates/foils^41,42^, and their data are also summarized in Fig. 1c for comparison. It can be found that the W heavy alloys exhibit higher elongation due to the addition of low-melting elements, which, however, will limit the high-temperature properties and applications of W-based alloys to some degree. Besides, W foil and W plate prepared through a proper rolling deformation possess a high UTS of ~1.8 GPa and a TE > 4% at room temperature, which is better than the present cWY alloy. However, the ultrathin thickness of W foil/plate limits their industrial applications. With the temperature increasing, the strength of cWY alloy decreases gradually while its plastic elongation increases at first and then decreases and peaks at 200 °C. The same experimental phenomenon was also found in other works^27^. However, as observed in Supplementary Fig. 2, our cWY alloy still shows better UTS (722 ± 10 MPa) and comparable TE (13.45 ± 1.09%) at 600 °C when compared with the congeneric W-Y_2_O_3_ alloys reported in literatures^26–30,43,44^, suggesting its increase in strength is not at the expense of ductility at high temperature. In order to provide more evidence of the mechanisms responsible for the reduced ductility, the fractographs of failed tensile specimens at different temperatures are shown in Supplementary Fig. 3. It can be seen that transgranular cleavage of W grains is the predominant fracture mode of cWY alloy below 200 °C (see Supplementary Fig. 3a–c), which is significantly different from the intergranular rupture mode of W grains in traditional WY alloy at low temperature (see Supplementary Fig. 4a–c). Therefore, W-W interfaces are strengthened greatly in cWY alloy when comparing with the traditional WY alloy, which could effectively promote the ductilizing of the developed alloy. With the increase of test temperature, ductile avulsion and interface rupture of W grains with the lamellar structure gradually dominate the fracture mode of cWY alloy (see Supplementary Fig. 3d), and this fracture mode is exacerbated at 600 °C (see Supplementary Fig. 3e). Thus it follows that the reduced ductility of cWY alloy at high temperature is closely linked to the ductile avulsion and interface rupture of W grains. Besides, the ductile avulsion of W grain also appears in WY alloy at 600 °C (see Supplementary Fig. 4e), which also results in the reduced ductility of WY alloy (see Supplementary Fig. 1).

### Characterization of oxide second-phase particle

**Fig. 2:**
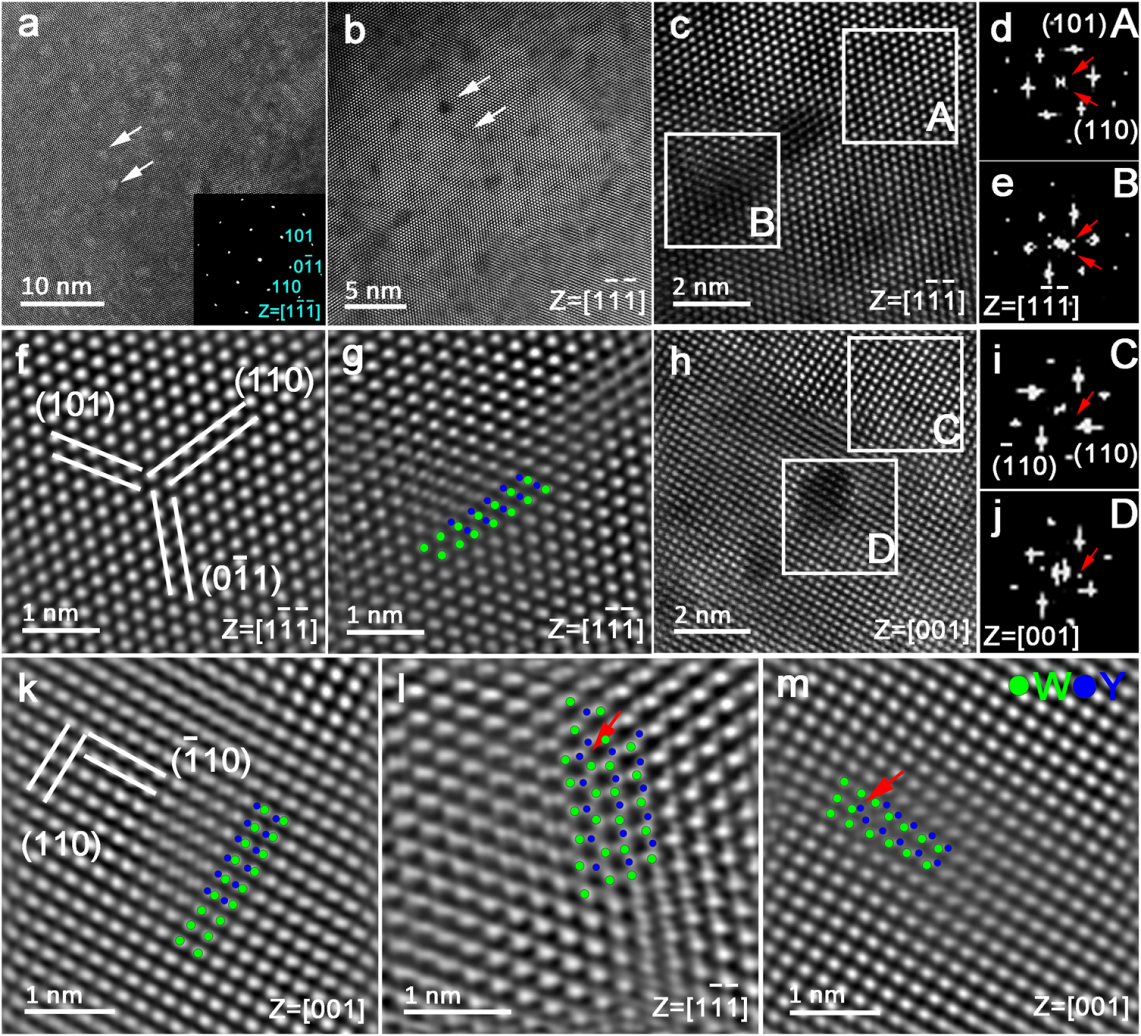
TEM and HAADF STEM images of intragranular oxide nanoparticles. **a, b** Bright-field TEM and HAADF STEM images taken from 〈111〉 zone axis reveal the existence of intragranular oxide nanoparticles (indicated by white arrows). The inset in **a** shows the SAED pattern from the corresponding microstructure. **c** Atomic-resolution HAADF STEM image showing a few coherent oxide nanoparticles. **d, e** The fast Fourier transform (FFT) patterns taken from W matrix and oxide nanoparticle, respectively, revealing the presence of Y columns along the {110} plane of W matrix. **f, g** Close-up images of region A and B in **c**, respectively, highlighting the periodic atomic columns in oxide nanoparticles. **h** HAADF STEM image taken from 〈001〉 zone axis showing the coherent oxide nanoparticle. **i, j** The FFT patterns are taken from **h**, revealing the presence of Y columns along the {110} plane of the W matrix. **k** Close-up image of region D showing the periodic atomic columns in oxide nanoparticles. **l**, **m** HAADF STEM images taken from 〈111〉 and 〈001〉 zone axis, respectively, showing another atomic configuration in oxide nanoparticles (denoted by red arrows). W and Y atoms are denoted as green and blue circles, respectively, and superimposed on **g**, **k**, **l**, and **m**.

In order to reveal the underlying mechanism behind this anomalous strengthening-toughening effect in the cWY alloy prepared in our work, the detailed microstructure prepared through our strategy is investigated. The secondary electron SEM (SE-SEM) image of the fracture surface of as-sintered cWY alloy (without HERF processing) is presented in Supplementary Fig. 5a. It can be seen that no oxide second-phase particles are distributed at W grain boundaries, suggesting that the oxide second-phase particles in cWY alloy are all dispersed within the W grain interior. This oxide distribution is dramatically different from that in the traditional WY alloys, where large-sized oxide second-phase particles always inevitably exist at W grain boundaries (see Supplementary Fig. 5b)^6,49,50^. Fig. 2a shows a representative high-resolution TEM (HRTEM) image of the W grain interior of the cWY alloy. Clearly, a very high number density of ultrafine spherical particles (1–3 nm) with white contrast are uniformly distributed within the W grain interior. The corresponding selected area electron diffraction (SAED) pattern from the 〈111〉_w_ zone axis (inset in Fig. 2a) reveals that the single-phase BCC structure of W has not been changed by these intragranular nanoparticles. But, the diffraction spots attributed to these second-phase nanoparticles are not detected. Next, the nanostructure of the cWY alloy down to atomic scale is further studied by high-angle annular dark-field (HAADF) STEM. Fig. 2b presents a HAADF STEM image taken from 〈111〉_w_ zone axis and the {110} crystal planes can be imaged. In this case, dark contrast originating from low atomic number (Z) can be observed. The dark contrast in HAADF imaging reveals the presence of regions enriched in light atoms (i.e., (Y, O)-rich), as denoted by white arrows in Fig. 2b. However, no sharp interfaces can be observed between these nanoparticles and the matrix, which seems to indicate a highly coherent interface relationship between them. Combining the results in Fig. 2a, b, it can be preliminarily demonstrated that the adscititious second-phase nanoparticles have been successfully introduced within the W grain interior. In Fig. 2c, nanoparticles are further viewed along the 〈111〉_w_ zone axis via an atomic-resolution HAADF STEM, and extra diffraction spots can be found in the corresponding fast Fourier transform (FFT) pattern (Fig. 2e). These diffraction spots should originate from the periodic array of Y or O columns. To observe the atomic columns of nanoparticles more clearly, the high-magnification HAADF STEM images from the W matrix and nanoparticles are shown in Fig. 2f, g, respectively. The regular intensity variations along W columns indicate Y atoms are arranged on the (101) crystal plane of the W matrix. A Y atom is located between two adjacent W atoms but the arrangement mode of W atoms is not changed along both (110) and (0–11) planes, forming the coherent interface with the surrounding W matrix. To further characterize the atomic structure of nanoparticles, we continue to take the HAADF image along 〈001〉 direction (see Fig. 2h). The weak spots assigned to the periodic array of Y columns again appear along the (110) crystal plane of the W matrix in the corresponding FFT pattern (Fig. 2j). From the corresponding high-magnification image (Fig. 2k), it can be found that Y atoms are located on the (−110) crystal plane of the W matrix. Two combined atoms (W and Y) in nanoparticles align with the W columns of the matrix along the (110) plane, forming the coherent interface between them. The W atoms and Y atoms are supposed to be bonded together via O bridging. In addition, another arrangement mode of the atomic column is also found along both 〈111〉 and 〈001〉 zone axis. As observed in Fig. 2l, m, W columns are in line with Y columns, but the coherent interface relationship is not destroyed. The bonded W and Y atoms are quite obvious in Fig. 2l and the atomic ratio of W:Y seems to be 1:1. To sum up, the oxide second-phase nanoparticles in cWY alloy are actually W-Y-O ternary phase rather than Y_2_O_3_ in traditional Y_2_O_3_ dispersion strengthened W alloys^30,51^.

Further atom probe tomography characterization result of the cWY alloy is presented in Supplementary Fig. 6a, where red surfaces encompassing regions containing more than 0.8 at % of Y are superimposed on the point cloud, highlighting the existence of regions enriched in Y. With near-spherical shape, these regions exhibit a size of 1–3 nm, in good agreement with the observations in Fig. 2a, b. Further composition analyses of the second-phase nanoparticles selected in Supplementary Fig. 6b are summarized in Supplementary Fig. 6c. The results reveal that the chemical composition of nanoparticles analyzed is almost similar, and the atomic ratio of W and Y is about 1:1, well in consistent with the observations in Fig. 2. Besides, the atomic ratio of Y and O (about 1:2) exceeds the stoichiometric ratio of Y_2_O_3_, which might be associated with the strong rare-earth-oxygen interactions of Y^51,52^. The detailed crystal structure of these oxide second-phase nanoparticles needs to be further investigated in the near future.

### Characterization of grain microstructure

**Fig. 3:**
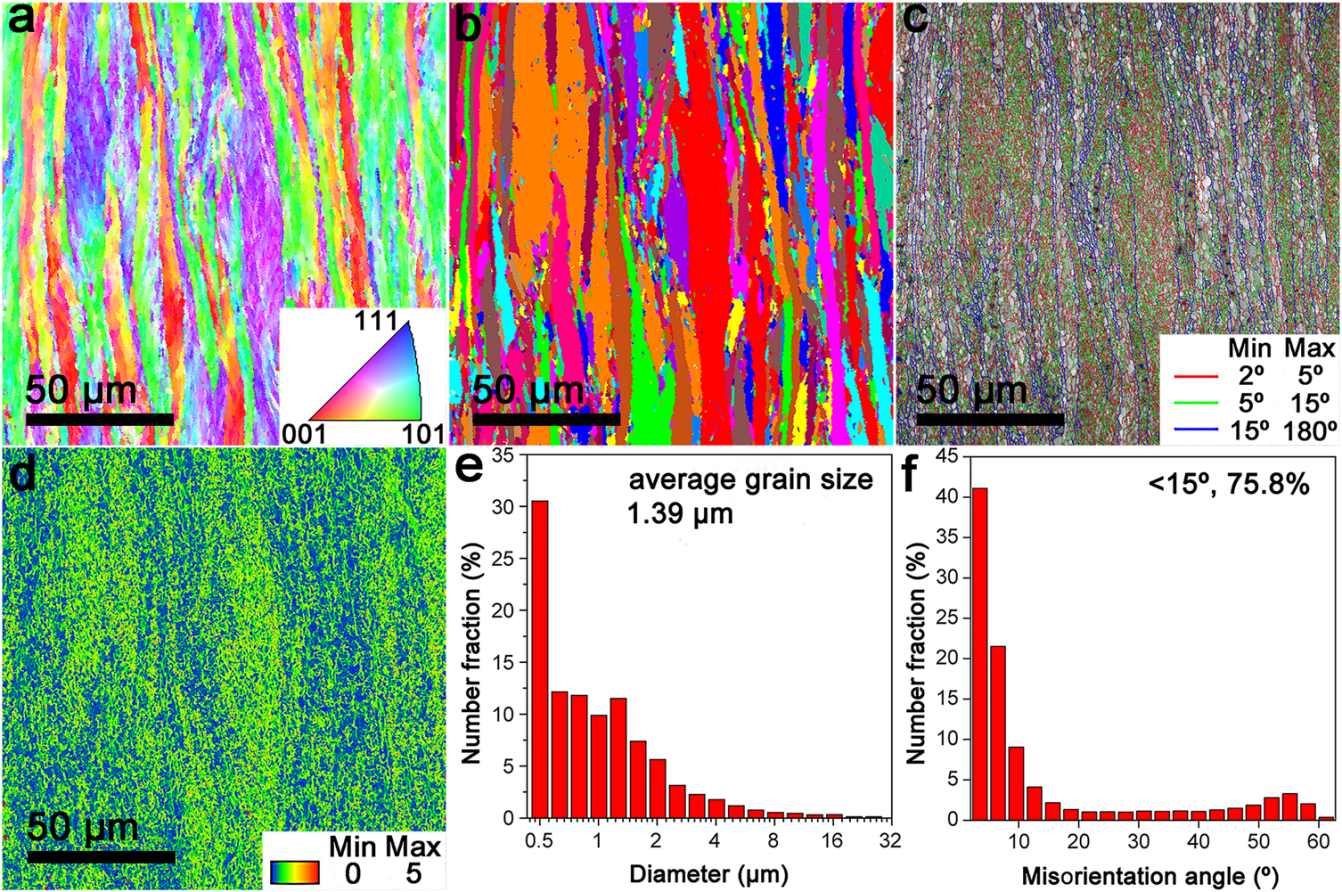
Electron back-scattering diffraction (EBSD) characterization of W grains. **a** Inverse polar figure (IPF) map of cWY alloy showing W grains along the rolling direction are not preferentially oriented. **b** Corresponding grain map revealing most of W grains are strongly elongated and coarsened after HERF processing. **c** Grain boundary distribution map revealing a combination structure of small-sized sub-grains existing in elongated mother-grains in cWY alloy. **d** Kernel average misorientation (KAM) map revealing that the larger the grains are, the more defect density or the greater plastic deformation they possess. **e** Grain size distribution image and **f** Grain boundary misorientation distribution image of cWY alloy.

Except for the distribution of oxide second-phase particles, the microstructural feature of the W matrix is another important factor affecting the mechanical property of cWY alloy. Fig. 3 is the electron back-scattering diffraction (EBSD) characterization of W grains taken from the rolling direction of cWY alloy. No strongly preferred orientation can be found for these W grains along the rolling direction through the inverse polar figure (IPF) map (Fig. 3a). But, most W grains are strongly elongated after HERF processing, as shown by the corresponding grain map (Fig. 3b). The average grain length/width of these lamellar grains is 17.8/2.3 μm, corresponding to an aspect ratio of about 8/1 (see the statistic result in Supplementary Fig. 7). Compared with the grain microstructure of as-sintered cWY alloy (average grain size of 1.3 ± 0.2 μm, see Supplementary Fig. 5a), it can be found that significant grain growth occurred on the cWY alloy during the HERF processing. The grain boundary distribution map (Fig. 3c) indicates that these elongated grains all have large misorientation angles (θ > 15°), as denoted by the blue lines. Meanwhile, the red and green lines (θ < 15°) further illustrate that these grains actually are composed of fine equiaxed sub-grains, which suggests dynamic recovery/recrystallization also occurred on these deformed grains during the HERF processing^27,30^. If the grains with a misorientation angle θ < 2° are measured, the average grain size can be calculated to be 1.39 μm (see Fig. 3e). From the grain boundary misorientation distribution image (Fig. 3f), it can be found that most of the grain boundaries of cWY alloy are low-angle grain boundary, further indicating extensive dynamic recovery/recrystallization were introduced by the HERF technique. Fig. 3d is the kernel average misorientation (KAM) map of the cWY alloy, revealing that the larger the grains are, the more defect density or the greater plastic deformation they possess. Thus, it follows that deformation and dynamic recovery/recrystallization are concomitant in our cWY alloy. In detail, a certain amount of dislocations introduced by deformation can be rearranged under the driving force of high temperature, during which the dislocations will undergo polygonization and further transform to subgrain boundary, then to fine-grained structure^53,54^. The grain microstructure in our cWY alloy is quite different from that in traditional W-Y_2_O_3_ alloys (see Supplementary Fig. 4), which generally have small-sized grains with minor dynamic recovery/recrystallization^30^. This can be attributed to the significantly different second-phase distribution in different alloys. For the traditional W-Y_2_O_3_ alloys, the intergranular oxide particles have a strong blocking effect on grain boundary migration, promoting grain stability at high temperatures. However, as characterized above, the oxide nanoparticles in cWY alloy are all dispersed within the W grain interior. Therefore, their blocking effect on the grain boundary migration is disappeared at high temperature, leading to severe grain growth and then high deformation level after HERF processing. To sum up, the small-sized sub-grains existing in elongated mother-grains and the optimized oxide second-phase particles distribution (nanoscale size, highly dispersed distribution within W grain interior, and fully coherent with W matrix) are two important microstructural features of cWY alloy.

## Discussion

### Preparation of oxide@W core-shell precursor powder

Generally speaking, the distinctive microstructures of cWY alloy, to a large extent, arise from its corresponding precursor powder, i.e., the oxide@W core-shell composite powder. Therefore, its unique preparation technology is the prerequisite for achieving the excellent performance of cWY alloy. Here, we develop a facile approach combining low-temperature hydrothermal and subsequent freeze-drying to realize this desirable powder structure. As for the traditional preparation methods, whether the mechanical alloying or co-deposited chemical method, the adscititious oxide particles generally attach to W powders only through physical binding, and the two phases are distinctly separated in the as-mixed state^55^. In this case, oxide nanoparticles located between W grains are easy to coalesce due to their aggregation effect at high temperature, leading to the relatively large intergranular oxide particles in the as-prepared W-based ODS alloys^30,56^. In contrast, if the oxide second-phase particles could be enclosed within the W grain interior in precursor powder, their self-aggregation will be restricted greatly, and highly dispersed intragranular nanoparticles will be introduced after subsequent sintering^10,30^. For this purpose, first and foremost, oxide nanoparticles with a uniform size distribution, excellent dispersancy, and controllable surface capping properties are urgently needed because this type of oxide could ensure all the nanoparticles to be homogeneously encapsulated within W grains through subsequent chemical reaction^57,58^. In this work, PVP-SDS diblock copolymer micelles are used as templates to guide and stabilize the formation of Y(OH)_3_ core nanoparticles with high monodispersity. Supplementary Fig. 8a shows the TEM image of synthesized Y(OH)_3_ nanoparticles. With near-spherical morphology and homogeneous size distribution (2–5 nm), these Y(OH)_3_ nanoparticles are the ideal precursor material of Y_2_O_3_ for the preparation of core-shell composite powder. Supplementary Fig. 8b is the HRTEM image of Y(OH)_3_ nanoparticles. The interplanar spacing of 0.216 and 0.186 nm can be assigned to the (201) and (300) crystal planes of Y(OH)_3_, respectively. After obtaining the Y(OH)_3_ core, another challenge in preparing core-shell precursor powder lies in the epitaxial growth of a completely different shell material (tungstate) on Y(OH)_3_ nanoparticles. Here, we dexterously adopt an acidic and water-soluble tungstate, i.e., ammonium metatungstate (AMT), to decorate the alkaline Y(OH)_3_ nanoparticles. Once it is added into the suspension of Y(OH)_3_, the (NH_4_)_18_Y_2_(W_12_O_40_)_3_ can be formed firstly via a neutralization reaction between Y(OH)_3_ and AMT. Subsequently, (NH_4_)_8_(W_12_O_40_) is induced to nucleate heterogeneously on (NH_4_)_18_Y_2_(W_12_O_40_)_3_ due to their similar chemical composition, forming an (NH_4_)_18_Y_2_(W_12_O_40_)_3_@(NH_4_)_8_(W_12_O_40_) core-shell structure. Supplementary Fig. 9a is the TEM image of the tungstate-encapsulated (NH_4_)_18_Y_2_(W_12_O_40_)_3_ particles and they exhibit a relatively large size compared with Y(OH)_3_ nanoparticles. From the corresponding HRTEM image (Supplementary Fig. 9b), it is clear that a few crystalline (NH_4_)_18_Y_2_(W_12_O_40_)_3_ nanoparticles with a size of about 5 nm are encapsulated within amorphous (NH_4_)_8_(W_12_O_40_). Next, with the presence of tungstate-encapsulated (NH_4_)_18_Y_2_(W_12_O_40_)_3_ as seeds, the freeze-drying method is used to drive the residual tungstate in solution to crystallize on their surfaces. After the subsequent reduction in a hydrogen atmosphere, the core is transformed into W-Y-O ternary oxide and the shell is transformed into W, forming oxide@W composite powder.

**Fig. 4:**
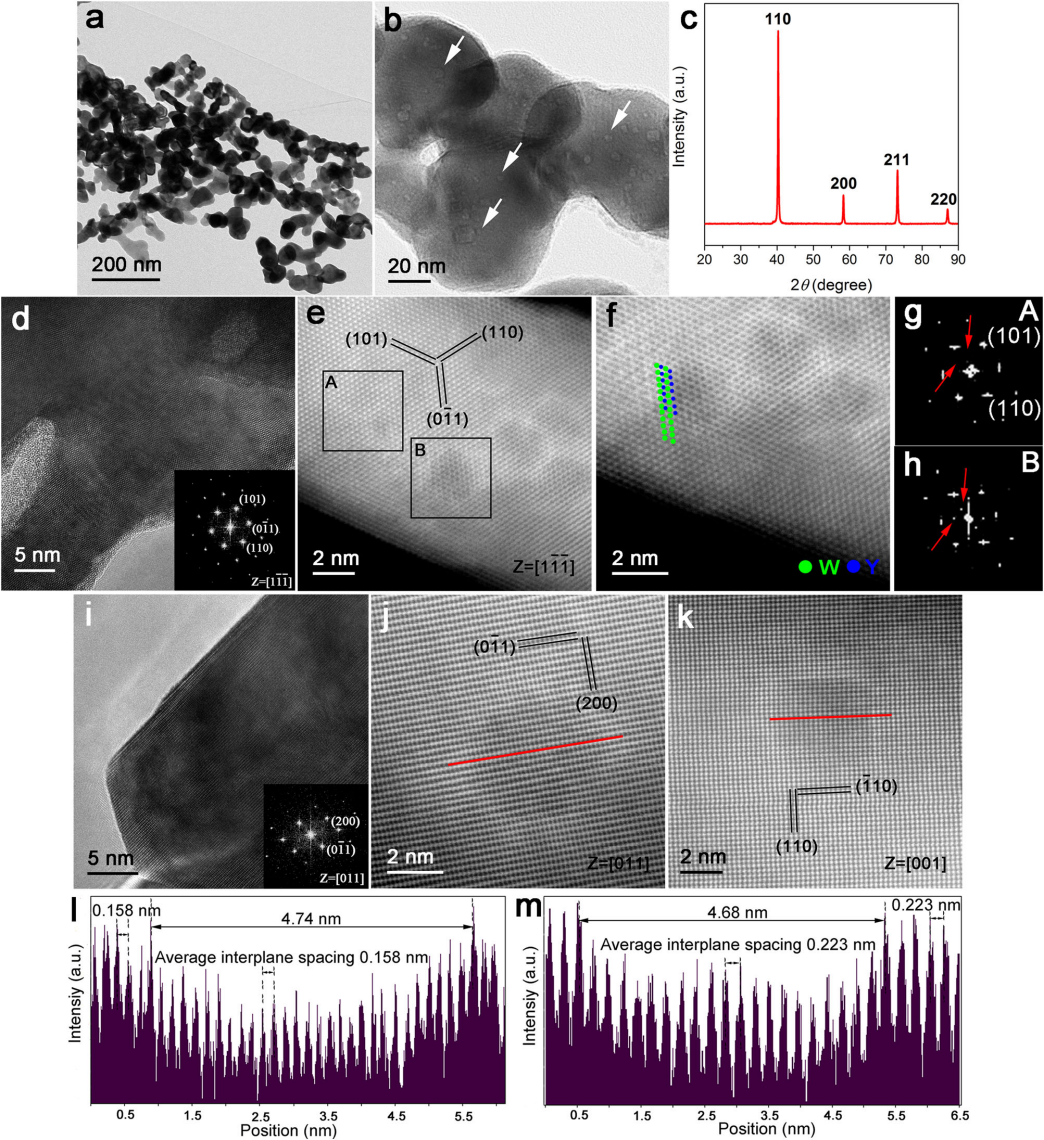
XRD, TEM, and HAADF STEM image of oxide@W core-shell structural composite powder. **a** TEM image of composite powder showing its homogeneous size distribution. **b** TEM image revealing the distribution of intragranular oxide nanoparticles (indicated by white arrows). **c** XRD pattern of core-shell composite powder. **d** HRTEM image of a powder grain taken from 〈111〉 zone axis revealing the highly coherent interface between oxide nanoparticle and W matrix The inset shows the SAED pattern of the corresponding microstructure. **e** HAADF STEM image showing a few coherent oxide nanoparticles. **f** The enlarged image of **e** highlighting the periodic atomic columns in oxide nanoparticles. W and Y atoms are denoted as green and blue circles, respectively, and superimposed on **f**. **g, h** The FFT patterns taken from the W matrix and oxide nanoparticles, respectively, revealing the presence of Y columns along the {110} plane of the W matrix. **i** HRTEM image of a powder grain taken from 〈011〉 zone axis similarly revealing the coherent interface relationship between oxide nanoparticles and W matrix The inset is the corresponding SAED pattern. **j**, **k** HAADF STEM images taken from 〈011〉 and 〈001〉 zone axis, respectively, revealing the presence of Y columns along the {110} plane of W matrix. **l**, **m** The intensity profiles taken along the red line in **j** and **k**, respectively. The periodic strong peaks on both sides represent W columns in the W matrix and the weak peaks lowered by Y atoms denote the W columns in oxide nanoparticles.

The typical microstructural characterization of prepared oxide@W core-shell composite powder is shown in Fig. 4. The TEM image (Fig. 4a) indicates that this unique powder with uniform size distribution has an average size of about 50 nm, which was calculated from more than 300 W grains. Besides, high-density oxide nanoparticles (2–5 nm) can be observed within the W grain interior from the corresponding HRTEM image (Fig. 4b). However, the peaks of the oxide phase are absent in the XRD pattern (Fig. 4c), which might be due to its small content or size. The pronounced four peaks are consistent with those of pure W with BCC structure, corresponding to the (110), (200), (211), and (220) planes. The average W grain size calculated using the Debye-Scherrer equation is 48.7 nm, well in consistent with the value obtained from the statistic analysis of the TEM result. Fig. 4d presents an HRTEM image of W grain taken from 〈111〉 zone axis. But, the crystal planes of W seem to continuously go through the whole region, suggesting that the coherent interface relationship between oxide nanoparticles and W matrix has formed as early as in the reduced composite powder. Characterized by atomic-resolution HAADF STEM, the BCC structure of W can be directly identified along 〈111〉 zone axis, as shown in Fig. 4e. The dark region therein can be ascribed to the intragranular oxide particles due to the Z-contrast reflected by HAADF STEM. Compared the FFT of region A (Fig. 4g) with that of region B (Fig. 4h), extra diffraction spots along (0–11) and (110) plane of the W matrix appear, which might be closely related to the presence of Y columns in region B. The related atomic columns are superimposed on the magnified HAADF STEM image in Fig. 4f, where W atoms in oxide nanoparticles have the same arrangement mode with that in the W matrix and Y atoms are arranged on the (101) crystal plane of W atoms, indicating the identical crystal structure of nanoparticles in composite powder and cWY alloy. In order to further investigate the occupancy of Y columns, the nanoparticles are also viewed along 〈011〉 and 〈001〉 zone axis, as shown in Fig. 4j, k, respectively. It can be seen that W atoms continue perfectly through the nanoparticles, further confirming their full lattice coherency with the W matrix. The existence of Y can be confirmed by the dark contrast along the {110} plane of the W matrix. The intensity profiles taken along the red line in Fig. 4j,k are shown in Fig. 4l, m, respectively. The periodic strong peaks on both sides represent W columns in the W matrix and the weak peaks lowered by Y atoms denote the W columns in oxide nanoparticles^59^. The interplanar spacings of both (200) and (110) planes have hardly been changed by the presence of Y columns. The resultant lowered elastic misfit between oxide nanoparticles and W matrix, in conjunction with the blocking effect of W matrix on atomic diffusion of oxide particles, are the important reasons why the coarsening of intragranular oxide nanoparticles can be greatly limited even at high temperature, as characterized in Fig. 2a, b.

### Strengthening and ductilizing mechanisms

In general, the mechanical properties of materials largely depend on their microstructural state. Specifically, for the cWY alloy developed in this work, its high strength mainly originates from the high-density intragranular nanoparticles and small-sized sub-grains, and its excellent ductility is closely associated with the strongly deformed microstructures with high fractions of small-angle grain boundaries and the intragranular nanoparticles that facilitate the generation and accumulation of dislocations within W grains interior rather than initiate cracks along grain boundaries. As mentioned above, optimized oxide particles (ultrafine size, coherent with W matrix, and highly dispersed distribution within W grain) are an important microstructural feature of cWY alloy. The interaction mechanism between the dislocations and the oxide nanoparticles can be either particle shearing or Orowan looping, which depends on the property and size of nanoparticles^11^. Considering that the oxide size (1–3 nm) of cWY alloy is far smaller than the critical size for Orowan looping, the gliding dislocations would shear these nanoparticles rather than loop them^20^. Therefore, the strengthening mechanism of intragranular oxide particles has transformed into dislocation shearing for cWY alloy in this work from Orowan looping for traditional W-based ODS alloys reported in the previous studies^6,10^. In those traditional W-based ODS alloys, the intragranular oxide particles with relatively large size and incoherent interface have weaker strengthening effects compared with the coherent nanoparticles. In our case, three factors that could contribute to coherent nanoparticle strengthening include ordering strengthening, modulus mismatch strengthening, and coherency strengthening^11,14,20^. The contributions of the first two factors to yield strength are estimated to be 463 MPa and 158 MPa, respectively (see the details in Supplementary Note 1). Therefore, if ignoring the smaller strengthening contribution from coherency strain, the total strengthening contribution of oxide nanoparticles is around 621 MPa. In addition, the increased grain boundary areas introduced by the sub-grain structure could serve as obstacles for moving dislocations, which is also conducive to the enhancement of material strength^30^. The contribution from grain boundary strengthening is estimated to be 470 MPa (see details in Supplementary Note 1). Therefore, the total yield strength, as the sum of the contributions from each mechanism, amounts to 1091 MPa, well in agreement with the experimental yield strength of 1200 MPa.

As for the W-based ODS alloy, the factors affecting its low-temperature ductility include microstructure, dislocation structure, and dislocation mobility. In particular, the dislocation mobility in the corresponding microstructure is critical to understanding the improvement effect of thermomechanical processing on the ductility of cWY alloy at low temperatures. Firstly, HERF results in strong texture and a lamellar grain structure with high fractions of low-angle grain boundaries, as shown in Fig. 3. The crystallographic texture is conductive to controlling cleavage planes and the orientation of crack propagation. The low-angle grain boundaries can help the dislocations to move across such boundaries easily. Secondly, HERF increases the density of dislocations and dislocation sources (see Fig. 3d), which reduces the energy requirement for dislocation motion. Consequently, the ductility of cWY alloy can be improved greatly through thermomechanical processing.

Another important factor that ductilizes cWY alloy is the optimized oxide distribution. As mentioned above, the oxide nanoparticles in cWY alloy are completely dispersed within the W grain interior and fully coherent with the W matrix, which is different from traditional W-based ODS alloys. As a result, the crack initiation associated with large-sized intergranular oxide particles is suppressed greatly^6,30^. More importantly, these high-density intragranular oxide particles have a strong blocking effect on the dislocation motion based on the theoretical calculation of strengthening contribution above. During the very first stages of plastic deformation, the dislocation motion prevails. Once the dislocations encounter the oxide particles, they will cut the oxide particles to progress through the material. Due to the strong pinning effect of these high-density oxide particles, as the deformation continues, an increasing number of dislocations will be pinned and accumulated within the W grain interior, which gives rise to remarkable strain hardening of alloy and then delays the initiation of necking^6,10^. The excellent strain hardening behavior of cWY alloy can be confirmed by comparing the tensile curves of cWY alloy and WY alloy in Supplementary Fig. 1. The dislocation cutting and retention within the W grain interior can be clearly seen in Supplementary Fig. 10. In addition, the dislocation pinning ability of the intragranular oxide particle in cWY alloy is further analyzed through a semi-quantitative method (see details in Supplementary Note 2), and the result confirms the improving effect of intragranular oxide nanoparticles on the ductility of cWY alloy. Furthermore, the coherent interfaces between oxide particles and W matrix also contribute to plastic deformation of cWY alloy by promoting dislocations slip across the interfaces^10,60^.

In summary, through sintering a unique oxide@W core-shell structural composite nanopowder, we have successfully prepared high-performance oxide dispersion strengthened W-based alloy. Our innovative low-temperature hydrothermal method and subsequent freeze-drying method enable the formation of oxide@W core-shell nanopowders. After low-temperature sintering and HERF processing, high-density oxide second-phase nanoparticles (1–3 nm) with coherent interface with the surrounding matrix are homogeneously dispersed within the W grain interior. Besides, refined equiaxed sub-grains are also introduced into the W matrix. This hierarchical microstructure, consequently, breaks the brittle characteristic of traditional W-based ODS alloys or pure W at room temperature and enables a combination of high strength and good ductility in the prepared alloy. More importantly, this strategy could provide theoretical guidance for designing other ODS alloy systems to simultaneously their strength and ductility.

## Methods

### Reagent

The yttrium nitrate hexahydrate (Y(NO_3_)_3_·6H_2_O, 99.99%), ammonium metatungstate (AMT, (NH_4_)_6_H_2_W_12_O_40_·xH_2_O, 99.5%), sodium dodecyl sulfate (SDS, GC, >99.0%), polyvinylpyrrolidone (PVP, (C_6_H_9_NO)_n_, K13-K18) and ammonia solution (NH_3_·H_2_O, 25–28%, GR) were all purchased from Shanghai Aladdin Company (Shanghai, China) and used as received without further purification. Deionized water was used as solvent in this experiment.

### Synthesis of Y(OH)_3_ suspension

In this work, the monodispersed Y(OH)_3_ nanoparticles produced by a facile hydrothermal method were used as core materials to prepare oxide@W composite powder precursor. During subsequent thermal processing, Y(OH)_3_ will transform into strengthening phase particles enriched in Y and O for the W matrix. In detail, yttrium nitrate hexahydrate was firstly dissolved in deionized water by ultrasonic treatment and then SDS and PVP were added in the as-obtained yttrium nitrate solution and stirred on a magnetic stirrer to get a completely transparent solution. The weight ratio of Y(NO_3_)_3_·6H_2_O, SDS, and PVP is about 1:3.5:1.25. Then, ammonia water was added to adjust the pH value of the mixing solution to about 10. The solution was then placed in a Teflon-sealed autoclave, and heated to 80 °C, and kept stirring for 6 h in an oil bath. After the autoclave was cooled to room temperature naturally, the as-prepared Y(OH)_3_ suspension with a color of light blue was taken out for the following experiments.

### Preparation of core-shell precursor powder

After obtaining the desired Y(OH)_3_ core, a controllable chemical reaction was first used to induce the epitaxial growth of tungstate onto the existing core nanoparticles. Then, in order to produce core-shell precursor powder, the freeze-drying method was adopted to facilitate the heterogeneous nucleation and growth of residual tungstate on the encapsulated seeds. Briefly, a certain amount of AMT was added in the as-prepared Y(OH)_3_ suspension at room temperature. With the aid of ultrasonic treatment, AMT will dissolve into the suspension swiftly. Then the suspension was subjected to vigorous stirring to achieve the complete chemical reaction between AMT and Y(OH)_3_ nanoparticles, turning the color of suspension from light blue to white. Thereafter, the white suspension was frozen quickly in liquid nitrogen to hinder the deposition of encapsulated seeds, which provides convenience for subsequent heterogeneous nucleation of dissolved tungstate onto each encapsulated seed particle. Finally, lyophilization was carried out for the prefrozen suspension in a freeze dryer to get core-shell precursor powder, which was then ground for later use.

### Thermal processing and sintering

In order to obtain composite powder for sintering W-based ODS alloy, the as-prepared core-shell precursor powder need to be thermal processed (calcination and reduction). It was firstly calcined at 480 °C for 1 h in an air atmosphere to fully remove impurity carbon from surfactants and macromolecular compounds and then reduced at 600 °C for 2 h and 800 °C for 2 h in a hydrogen atmosphere. Finally, the resultant composite powder was cooled to room temperature in flowing hydrogen and stored in a glove box for subsequent sintering. The sintering of W-based ODS alloy was implemented in an atmosphere furnace. Firstly, the composite powder was squeezed into a cylindrical compact with a diameter of 36 mm and a height of 14 mm via cold isostatic pressing. Then the pressed compact was sintered at 1650 °C for 4 h in flowing hydrogen. Subsequently, the as-sintered cylindrical compact was thermo-mechanically processed into a flat disc with a diameter of 65 mm and a high of 4 mm via high-energy-rate forging (HERF) at 1550 °C. Finally, the disc was annealed at 1100 °C for 30 min in a hydrogen atmosphere to relieve residual stress and stabilize the microstructure. This final prepared W-based ODS alloy using the oxide@W core-shell composite powder as a precursor is denoted as cWY alloy.

### Atom probe tomography

An instrument called Cameca LEAP 3000X HR was employed to carry out the atom probe tomography analysis for the cWY alloy. The related test conditions include an ultrahigh vacuum of about 2.5 × 10^−11^ torr, an ultralow test temperature of 80 K, and a target evaporation rate of 3 ions for 1000 pulses on average in high-voltage pulsing mode at a 15% pulse fraction. A dual-beam FEI Helios 600 was used to prepare the specimens for the test via the focused ion beam milling. The CAMECA integrated visualization and analysis software IVAS 3.6.8 was used for three-dimensional atomic reconstruction and data processing.

### Mechanical properties test

The tensile tests were conducted on an Instron 5967 machine in the air at a constant loading rate of 0.06 mm/min. The dog-bone-shaped specimens with a cross-section of 1.5 × 0.8 mm^2^ and a working length of 5 mm were machined from the cWY alloy along the flat surface. Before tests, all tensile specimens were mechanically polished. The test temperature was varied from room temperature to 600 °C.

### Characterization

The phase composition and microstructure of precursor powder, reduced composite powder and the sintered alloy were characterized by X-ray diffraction (XRD, D/MAX-2500) with Cu Kα radiation, field emission scanning electron microscopy (SEM, Hitachi model No. S 4800), and transmission electron microscopy (TEM, JEM-2100), respectively. High-angle annular dark-field (HAADF) STEM images were taken on a JEOL JEM-ARM200F instrument using an annular-type detector. The S/TEM specimens were firstly ground to 20 μm on SiC abrasive papers and then thinned on an ion beam thinner (Gatan-PIPS695). Electron backscatter diffraction pattern (EBSD) mappings of cWY alloys were collected using a field emission scanning electron microscopy (SEM, JSM-7800F) equipped with a CRYSTAL detector (NordlysMax^2^). The specimens for EBSD characterization were firstly ground on SiC abrasive papers. Then they were further polished on a metallographic lapping (UNIPOL-820) with the aid of diamond polishing spray, followed by electrolytic polishing in 5% sodium hydroxide aqueous solution with a constant voltage of 11 V and a current density of 3 mA/mm^2^.

## Supplementary information

Supplementary Information

## Data Availability

The data that support the findings of this work are available from the corresponding author upon reasonable request.

## References

[1] Lu L, Shen YF, Chen XH, Qian LH, Lu K (2004). Ultrahigh strength and high electrical conductivity in copper. Science.

[2] Li XY, Wei YJ, Lu L, Lu K, Gao HJ (2010). Dislocation nucleation governed softening and maximum strength in nano-twinned metals. Nature.

[3] Wang YM, Chen MW, Zhou FH, Ma E (2002). High tensile ductility in a nanostructured metal. Nature.

[4] Zhao YH (2008). High tensile ductility and strength in bulk nanostructured nickel. Adv. Mater..

[5] Zhao YH, Liao XZ, Cheng S, Ma E, Zhu YT (2006). Simultaneously increasing the ductility and strength of nanostructured alloys. Adv. Mater..

[6] Liu G (2013). Nanostructured high-strength molybdenum alloys with unprecedented tensile ductility. Nat. Mater..

[7] Fang TH, Li WL, Tao NR, Lu K (2011). Revealing extraordinary intrinsic tensile plasticity in gradient nano-grained copper. Science.

[8] Lee YK, Jin JE, Ma YQ (2007). Transformation-induced extraordinary ductility in an ultrafine-grained alloy with nanosized precipitates. Scr. Mater..

[9] Zan YN (2019). Enhancing strength and ductility synergy through heterogeneous structure design in nanoscale Al_2_O_3_ particulate reinforced Al composites. Mater. Des..

[10] Huang L (2017). In situ oxide dispersion strengthened tungsten alloys with high compressive strength and high strain-to-failure. Acta Mater..

[11] Jiao ZB, Luan JH, Miller MK, Yu CY, Liu CT (2015). Effects of Mn partitioning on nanoscale precipitation and mechanical properties of ferritic steels strengthened by NiAl nanoparticles. Acta Mater..

[12] Vieider G (1999). European development of the ITER divertor target. Fusion Eng. Des..

[13] Tjong SC, Ma ZY (2000). Microstructural and mechanical characteristics of in situ metal matrix composites. Mater. Sci. Eng. R..

[14] Wang Q (2018). Coherent precipitation and strengthening in compositionally complex alloys: a review. Entropy.

[15] Hu YS (2020). Simultaneous enhancement of strength and ductility with nano dispersoids in nano and ultrafine grain metals: a brief review. Rev. Adv. Mater. Sci..

[16] Zhao JC, Notis MR (1998). Spinodal decomposition, ordering transformation, and discontinuous precipitation in a Cu-15Ni-8Sn alloy. Acta Mater..

[17] Vissers R (2007). The crystal structure of the β‘-phase in Al-Mg-Si alloys. Acta Mater..

[18] Song GA, Sun ZQ, Poplawsky JD, Gao YF, Liaw PK (2017). Microstructural evolution of single Ni_2_TiAl or hierarchical NiAl/Ni_2_TiAl precipitates in Fe-Ni-Al-Cr-Ti ferritic alloys during thermal treatment for elevated-temperature applications. Acta Mater..

[19] Sha G, Cerezo A (2004). Early-stage precipitation in Al-Zn-Mg-Cu alloy (7050). Acta Mater..

[20] Jiang S (2017). Ultrastrong steel via minimal lattice misfit and high-density nanoprecipitation. Nature.

[21] Suryanarayana C, Al-Aqeeli N (2013). Mechanically alloyed nanocomposites. Prog. Mater. Sci..

[22] Kannan C, Ramanujam R (2017). Comparative study on the mechanical and microstructural characterisation of AA 7075 nano and hybrid nanocomposites produced by stir and squeeze casting. J. Adv. Res..

[23] Dong Z, Hu W, Ma Z, Li C, Liu Y (2019). The synthesis of composite powder precursors via chemical processes for the sintering of oxide dispersion-strengthened alloys. Mater. Chem. Front..

[24] Jiang L, Li ZQ, Fan GL, Cao LL, Zhang D (2012). The use of flake powder metallurgy to produce carbon nanotube (CNT)/aluminum composites with a homogenous CNT distribution. Carbon.

[25] Kai XZ (2013). Strong and ductile particulate reinforced ultrafine-grained metallic composites fabricated by flake powder metallurgy. Scr. Mater..

[26] Zhang T (2018). Recent progress of oxide/carbide dispersion strengthened W-based materials. Acta Metall. Sin..

[27] Lian Y (2017). Mechanical properties and thermal shock performance of W-Y_2_O_3_ composite prepared by high-energy-rate forging. Phys. Scr..

[28] Xie ZM (2015). Effect of high temperature swaging and annealing on the mechanical properties and thermal conductivity of W-Y_2_O_3_. J. Nucl. Mater..

[29] Liu R (2016). Nanostructured yttria dispersion-strengthened tungsten synthesized by sol-gel method. J. Alloy. Compd..

[30] Xie ZM (2016). Achieving high strength/ductility in bulk W-Zr-Y_2_O_3_ alloy plate with hybrid microstructure. Mater. Des..

[31] Xie ZM (2017). Recrystallization and thermal shock fatigue resistance of nanoscale ZrC dispersion strengthened W alloys as plasma-facing components in fusion devices. J. Nucl. Mater..

[32] Xie ZM (2015). Extraordinary high ductility/strength of the interface designed bulk W-ZrC alloy plate at relatively low temperature. Sci. Rep..

[33] Ding HL (2018). Determination of the DBTT of nanoscale ZrC doped W alloys through amplitude-dependent internal friction technique. Mater. Sci. Eng. A.

[34] Miao S (2016). Mechanical properties and thermal stability of rolled W-0.5wt% TiC alloys. Mater. Sci. Eng. A.

[35] Kumari A, Sankaranarayana M, Nandy TK (2017). On structure property correlation in high strength tungsten heavy alloys. Int. J. Refract. Met. Hard Mater..

[36] Durlu N, Çalişkan NK, Bor Ş (2014). Effect of swaging on microstructure and tensile properties of W–Ni–Fe alloys. Int. J. Refract. Met. Hard Mater..

[37] Kiran UR, Panchal A, Sankaranarayana M, Nandy TK (2013). Tensile and impact behavior of swaged tungsten heavy alloys processed by liquid phase sintering. Int. J. Refract. Met. Hard Mater..

[38] Kiran UR (2017). Refractory metal alloying: a new method for improving mechanical properties of tungsten heavy alloys. J. Alloy. Compd..

[39] Zhou S (2019). High entropy alloy: a promising matrix for high-performance tungsten heavy alloys. J. Alloy. Compd..

[40] Leonhardt T (2009). Properties of tungsten-rhenium and tungsten-rhenium with hafnium carbide. JOM.

[41] Reiser J (2013). Tungsten foil laminate for structural divertor applications—Tensile test properties of tungsten foil. Nucl. Mater..

[42] Wei Q, Kecskes LJ (2008). Effect of low-temperature rolling on the tensile behavior of commercially pure tungsten. Mater. Sci. Eng., A.

[43] Tan XY (2015). Mechanical properties and microstructural change of W-Y_2_O_3_ alloy under helium irradiation. Sci. Rep..

[44] Battabyal M (2013). Microstructure and mechanical properties of a W-2wt.%Y_2_O_3_ composite produced by sintering and hot forging. J. Nucl. Mater..

[45] Yan QZ, Zhang XX, Wang TN, Yang CT, Ge CC (2013). Effect of hot working process on the mechanical properties of tungsten materials. J. Nucl. Mater..

[46] Shen T, Dai Y, Lee Y (2016). Microstructure and tensile properties of tungsten at elevated temperatures. J. Nucl. Mater..

[47] Deng HW (2018). Mechanical properties and thermal stability of pure W and W-0.5wt%ZrC alloy manufactured with the same technology. Mater. Sci. Eng. A.

[48] Krsjak V, Wei SH, Antusch S, Dai Y (2014). Mechanical properties of tungsten in the transition temperature range. J. Nucl. Mater..

[49] Dong Z (2020). The simultaneous improvements of strength and ductility in W-Y_2_O_3_ alloy obtained via an alkaline hydrothermal method and subsequent low temperature sintering. Mater. Sci. Eng. A.

[50] Dong Z, Ma Z, Yu L, Liu Y (2020). Enhanced mechanical properties in oxide dispersion strengthened alloys achieved via interface segregation of cation dopants. Sci. China Mater..

[51] Dong Z (2017). Synthesis of nanosized composite powders via a wet chemical process for sintering high performance W-Y_2_O_3_ alloy. Int. J. Refract. Met. Hard Mater..

[52] Hu W, Dong Z, Wang H, Ahamad T, Ma Z (2021). Microstructure refinement and mechanical properties improvement in the W-Y_2_O_3_ alloys via optimized freeze-drying. Int. J. Refract. Met. Hard Mater..

[53] Zhao ZB, Wang QJ, Liu JR, Yang R (2017). Effect of heat treatment on the crystallographic orientation evolution in a near-α titanium alloy Ti60. Acta Mater..

[54] Gey N, Bocher P, Uta E, Germain L, Humbert M (2012). Texture and microtexture variations in a near-α titanium forged disk of bimodal microstructure. Acta Mater..

[55] Liu N (2019). Eliminating bimodal structures of W-Y_2_O_3_ composite nanopowders synthesized by wet chemical method via controlling reaction conditions. J. Alloy. Compd..

[56] Hu W, Kong X, Du Z, Khan A, Ma Z (2021). Synthesis and characterization of nano TiC dispersed strengthening W alloys via freeze-drying. J. Alloy. Compd..

[57] Kamata K, Lu Y, Xia YN (2003). Synthesis and characterization of monodispersed core-shell spherical colloids with movable cores. J. Am. Chem. Soc..

[58] Lu Y, Yin YD, Li ZY, Xia YN (2002). Synthesis and self-assembly of Au@SiO_2_ core-shell colloids. Nano Lett..

[59] Shibata N (2004). Observation of rare-earth segregation in silicon nitride ceramics at subnanometre dimensions. Nature.

[60] Lei ZF (2018). Enhanced strength and ductility in a high-entropy alloy via ordered oxygen complexes. Nature.

