# Supplementary material for: Achieving high strength and ductility in ODS-W alloy by employing oxide@W core-shell nanopowder as precursor
Source: Nat Commun. 2021 Aug 20;12:5052. doi: 10.1038/s41467-021-25283-2 (PMC8379241; doi:10.1038/s41467-021-25283-2)
Supplement: Supplementary file 1 — Supplementary Information [file 41467_2021_25283_MOESM1_ESM.pdf]

Supplementary Information

Achieving high strength and ductility in ODS-W alloy by employing oxide@W core-shell nanopowder as precursor

Dong et al.

Supplementary Figures

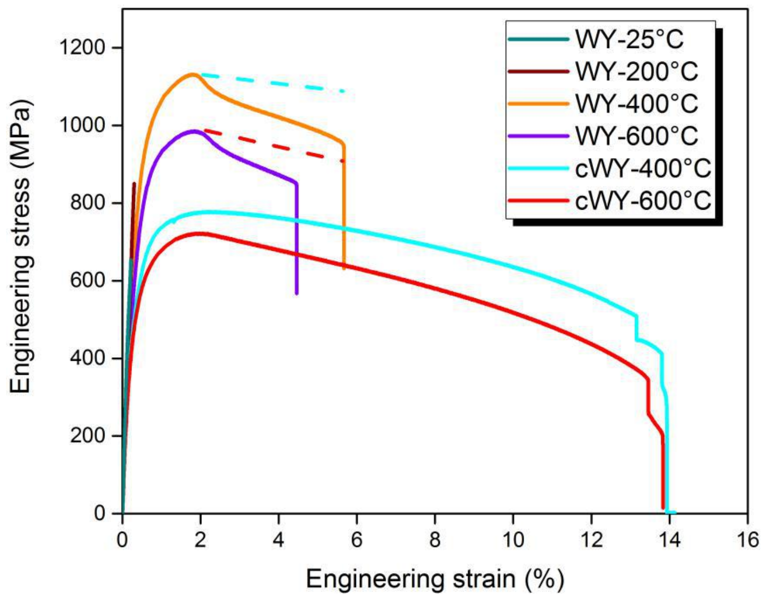

Supplementary Figure 1 Mechanical properties of WY alloy. Tensile curves of WY alloy tested at temperature from 25 to 600 ˚C. The tensile curves of cWY alloy tested at 400 ˚C and 600 ˚C are also plotted to highlight the strain hardening phenomenon in cWY alloy. The dotted lines on the tensile curves of WY alloy exhibit the same slope with the tensile curve of cWY alloy at small strains. The gaps between dotted lines and tensile curves confirm the enhanced strain hardening behavior of our cWY alloy.

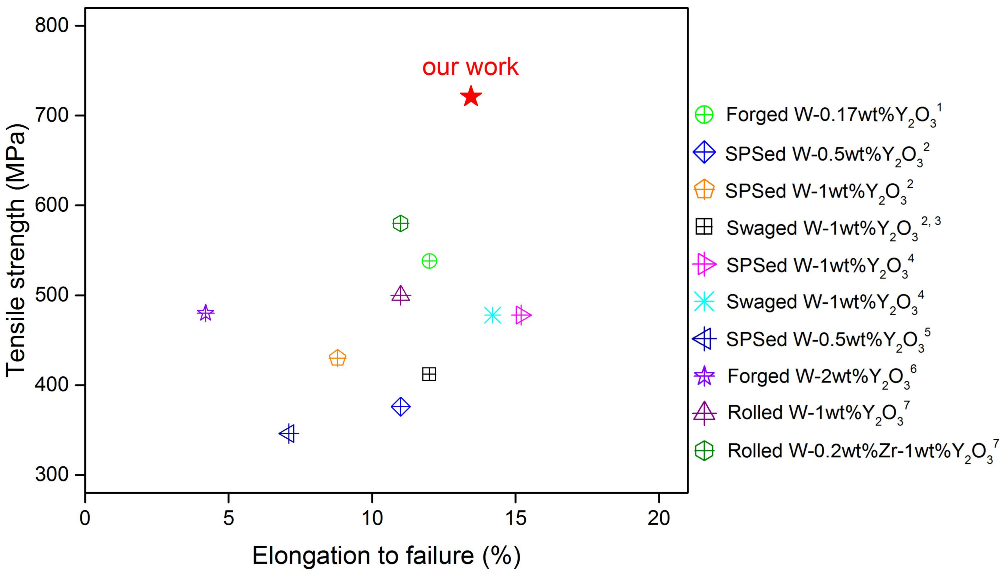

Supplementary Figure 2 Mechanical properties of cWY alloy at high temperature. Comparison of tensile strength and total elongation tested at 600 ˚C between the cWY alloy developed in this work and Y_2O_3 dispersion-strengthened W-based alloys reported in previous works[1-7].

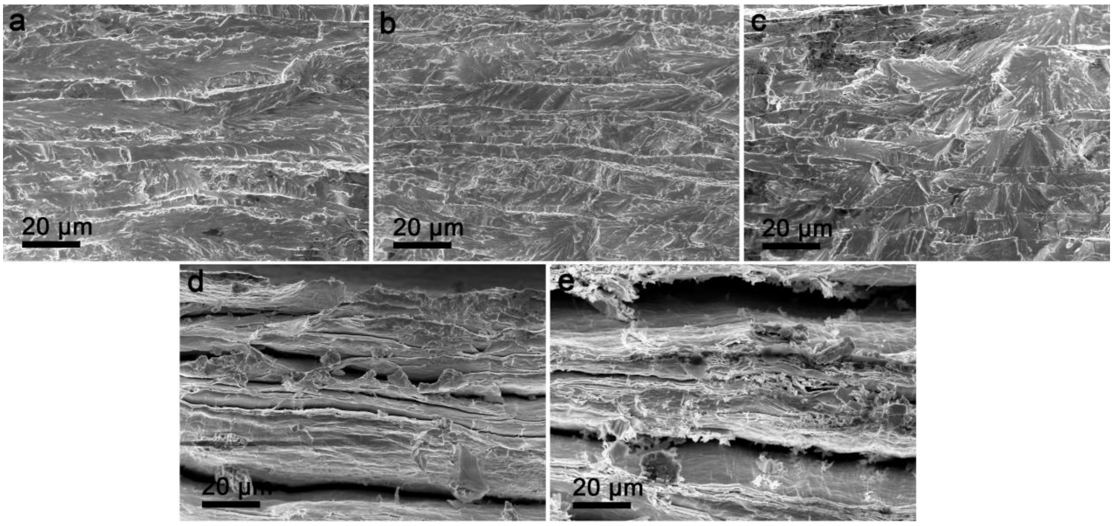

Supplementary Figure 3Fracture morphology of cWY alloy. Fractographs of failed tensile cWY specimen tested at **a** 25 ˚C. **b** 100 ˚C. **c** 200 ˚C. **d** 400 ˚C. **e** 600 ˚C.

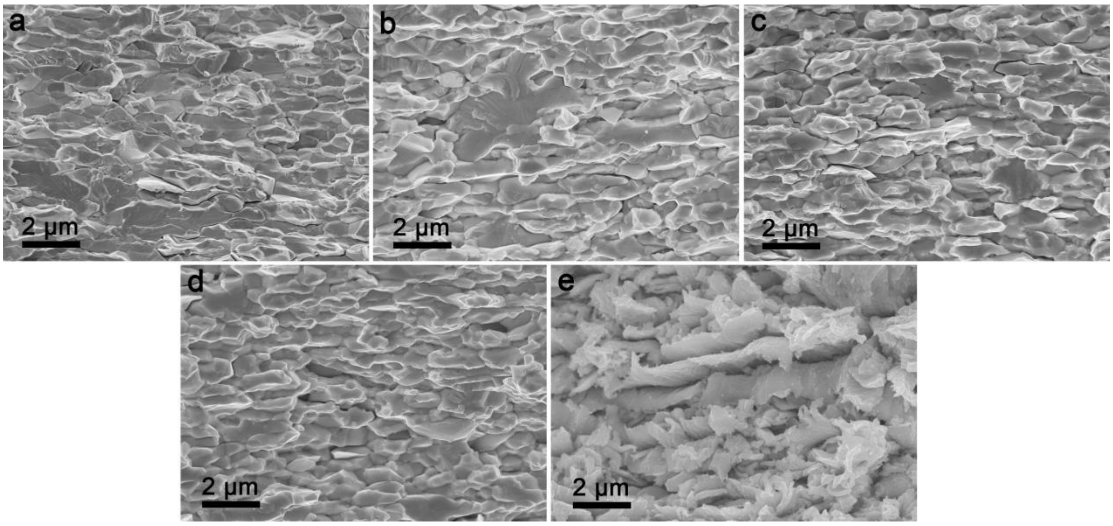

Supplementary Figure 4Fracture morphology of WY alloy. Fractographs of failed tensile WY specimen at **a** 25 ˚C. **b** 100 ˚C. **c** 200 ˚C. **d** 400 ˚C. **e** 600 ˚C.

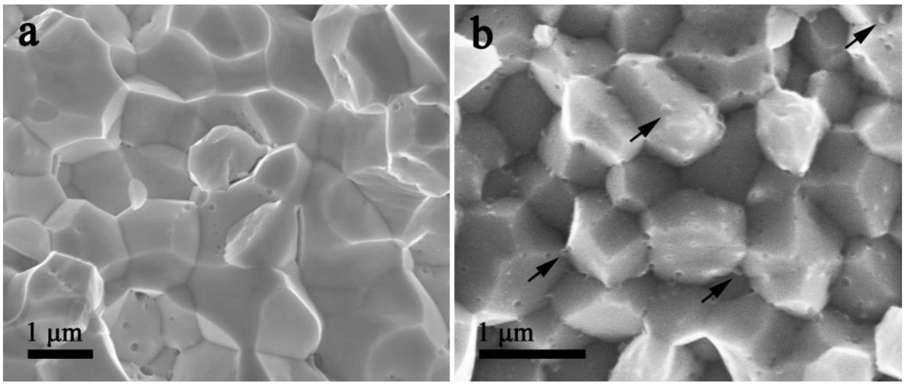

Supplementary Figure 5 Fracture morphology of sintered alloys.a Secondary electron SEM (SE-SEM) image of fracture surface of the as-sintered cWY alloy. **b**SE-SEM image of fracture surface of the as-sintered WY alloy, whose intergranular oxide particles are denoted by black arrows.

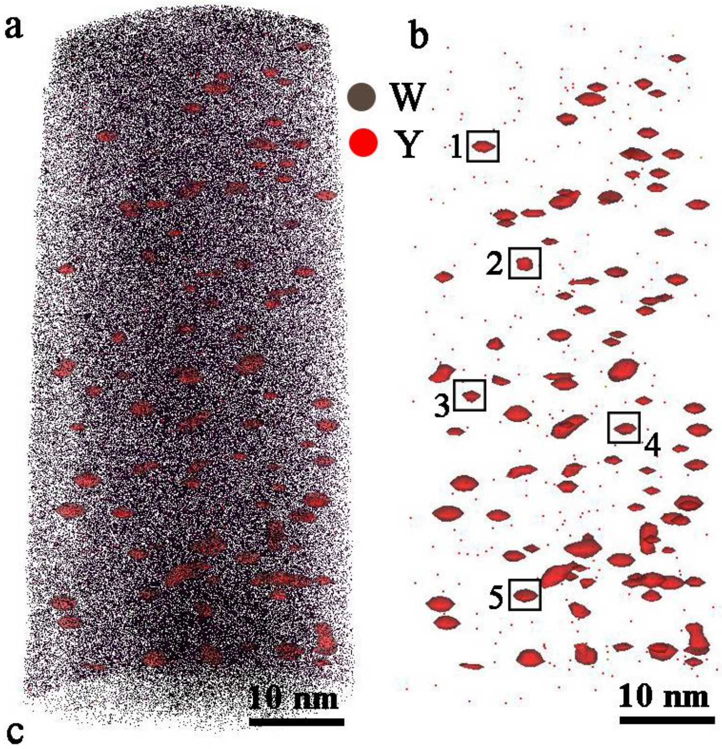

c

Particle No.	W (at.%)	Y (at.%)	O (at.%)
1	29.2	24.3	46.4
2	25.8	25.6	48.5
3	28.3	25.4	46.4
4	26.5	25.5	48.1
5	28.4	27.1	44.6
Average	27.6±1.2	25.5±0.9	46.8±1.3

Supplementary Figure 6 Chemical compositions of the oxide nanoparticles in the cWY alloy. **a** Atom probe tomography three-dimensional reconstruction from the analysis of the cWY alloy. The red surfaces encompassing regions containing more than 0.8 at% of Y are superimposed on the point cloud, highlighting the existence of regions enriched in Y. **b** The oxide nanoparticles selected for composition analyses. **c** Corresponding chemical composition.

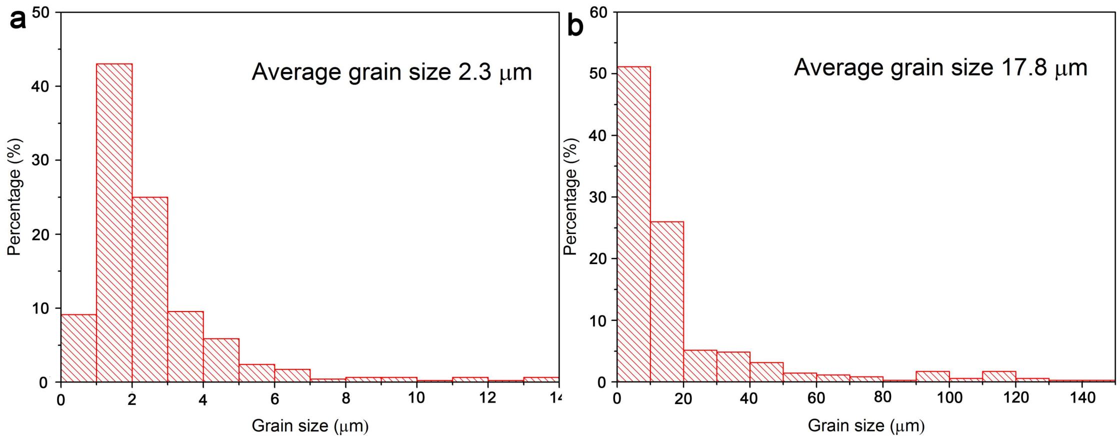

Supplementary Figure 7 The statistic result of grain size.a grain length and **b** grain width of the lamellar grains in cWY alloy by measuring about 500 grains.

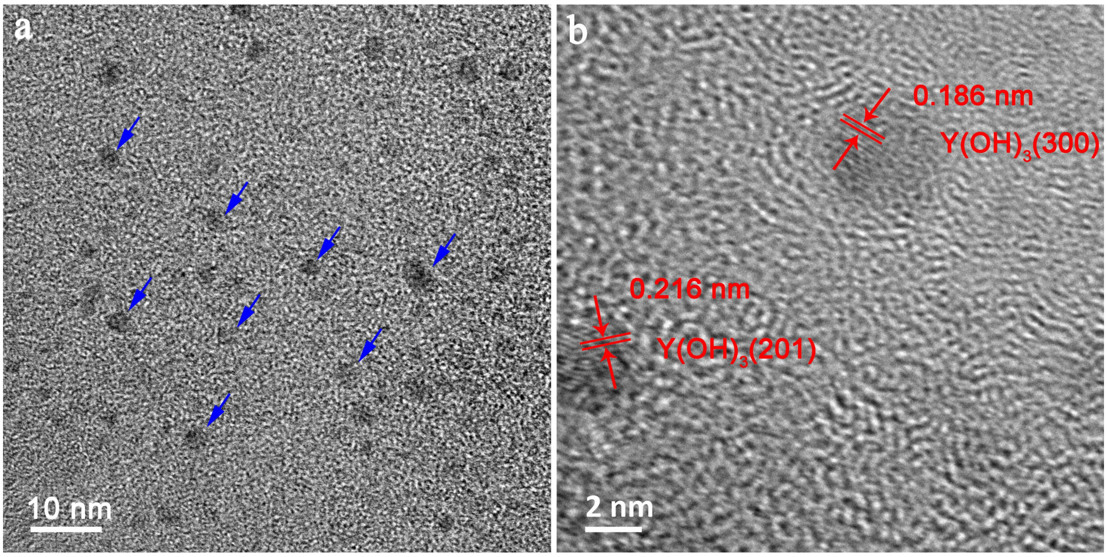

Supplementary Figure 8 TEM characterization of $Y(OH)_3$ nanoparticles.a TEM image of the synthesized $Y(OH)_3$ nanoparticles with a size of 2-5 nm (indicated by blue arrows). **b** HRTEM image of $Y(OH)_3$ nanoparticles. The interplanar spacing of 0.216 and 0.186 nm can be assigned to the (201) and (300) crystal planes of $Y(OH)_3$, respectively.

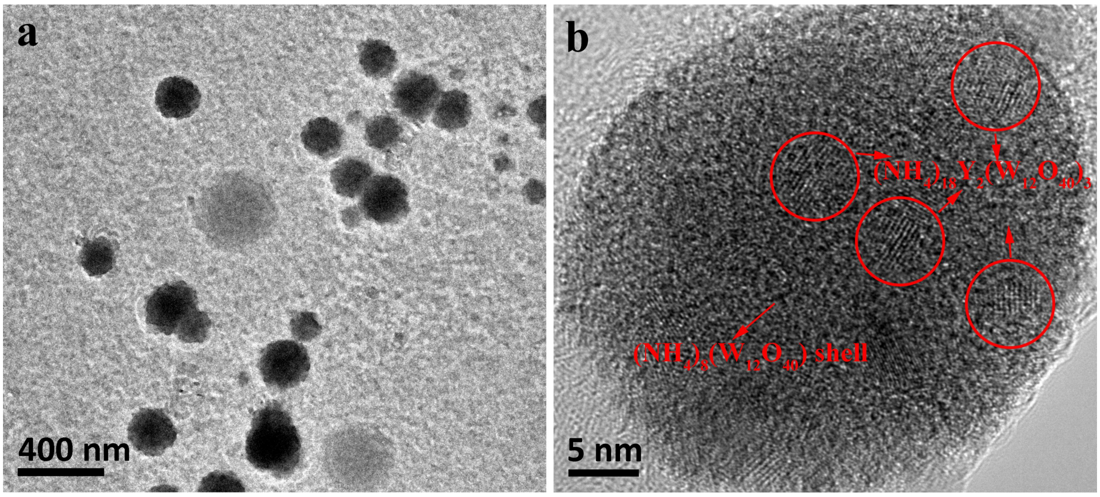

Supplementary Figure 9 TEM characterization of tungstate-encapsuled $(NH_4)_{18}Y_2(W_{12}O_{40})_3$ particles.a TEM image of the tungstate-encapsuled $(NH_4)_{18}Y_2(W_{12}O_{40})_3$ particles. **b** Corresponding HRTEM image showing that a few crystalline $(NH_4)_{18}Y_2(W_{12}O_{40})_3$ nanoparticles (indicated by red circles) with size of about 5 nm are encapsulated within amorphous $(NH_4)_8(W_{12}O_{40})$.

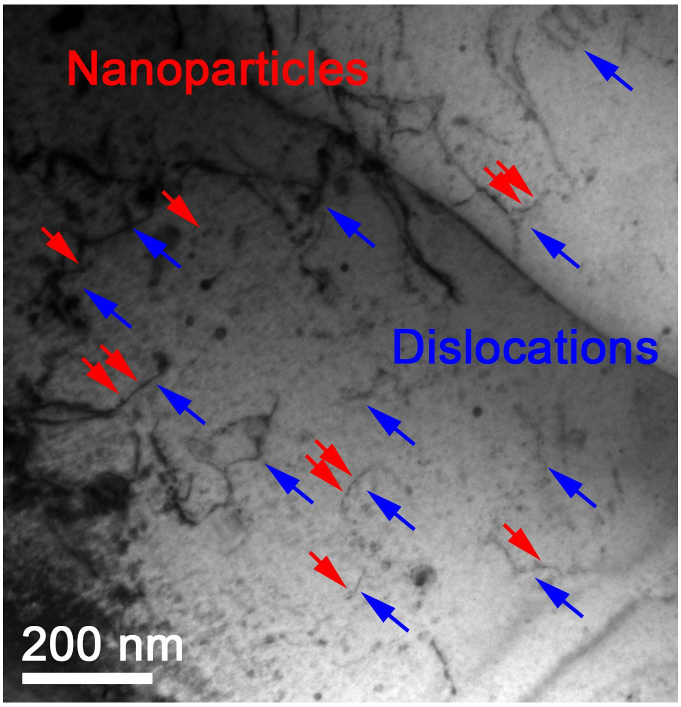

Supplementary Figure 10Characterization of dislocations. TEM image of the failed tensile cWY alloy at 25 ˚C, showing the interaction between oxide nanoparticles and dislocations and the retention of dislocations within W grain interior. The dislocations are indicated by blue arrows and intragranular oxide particles are indicated by red arrows

Supplementary Note 1

For the ordering strengthening, the strength increase can be expressed as[8]:

$$\Delta\sigma_{ordering} = \frac{M\gamma_{apb}^{3/2}}{b}\left(\frac{4r_s f}{\pi T_l}\right)^{1/2} \tag{1}$$

where M is the Taylor factor of 2.73, b = 0.27 nm is Burgers vector of dislocation of W, γ_{apb} is the antiphase boundary energy for the intragranular oxide nanoparticles, which can be calculated to be 0.99 J m^{-2} based on the atomic arrangement of oxide nanoparticle, f is the average volume fraction of oxide nanoparticles, r_s is the average radius of the sheared oxide nanoparticles in the gliding plane, T_l is the dislocation line tension, and can be approximated as $Gb^2/2$. G = 161 GPa is the shear modulus of W matrix. Then, $\Delta\sigma_{ordering}$ was calculated to be 463 MPa.

For the modulus mismatch strengthening, the strength increase can be described by[9]:

$$\Delta\sigma_{\mathrm{mod}ulus} = \frac{M\Delta G}{4\pi^2}\left(\frac{3\Delta G}{Gb}\right)^{1/2}\left[0.8 - 0.143\ln\left(\frac{r}{b}\right)\right]^{2/3} r^{1/2} f^{1/2} \tag{2}$$

where r is the average radius of oxide nanoparticles, ΔG is the shear modulus difference between oxide nanoparticles and W matrix. Based on the atomic composition and arrangement of oxide nanoparticle and W matrix, ΔG is set equal to the shear modulus of Y (26 GPa). Then, $\Delta\sigma_{\mathrm{mod}ulus}$ was calculated to be 158 MPa.

The grain boundary strengthening effect of subgrains in cWY alloy can be understood using Hall-Petch equation[10]:

$$\sigma_y = \sigma_0 + k_y D^{-1/2} \tag{3}$$

where σ_0 is the lattice friction stress and k_y is the Hall-petch constant. The value

of σ_0 and k_y were adopted from that of traditional W-Y_2O_3 alloy produced under the same process conditions. As a result, for the cWY alloy with a grain size of 1390 nm, σ_y is determined to be 470 MPa. Therefore, the total strength due to the oxide nanoparticle strengthening and grain refinement amounts to 1091 MPa, well in agreement with the experimental yield strength of 1200 MPa.

Supplementary Note 2

Here, in order to estimate the dislocation pinning ability of the intragranular oxide particle in cWY alloy, the increase rate of stored dislocation density is calculated according to eq 4[11], which is a semi-quantitative analysis of the improved ductility through optimizing oxide distribution.

$$\frac{d\rho}{d\varepsilon_p} = \frac{1}{\lambda_{\mathrm{int}ra}^2}\frac{dn_\perp}{d\varepsilon_p} = \frac{M}{b}\frac{d_{\mathrm{int}ra}}{\lambda_{\mathrm{int}ra}^2} = \frac{M}{d_{\mathrm{int}ra}b}\left(\frac{6f_{\mathrm{int}ra}}{\pi}\right)^{2/3} \tag{4}$$

where ρ is the stored dislocation density, ε_p is the resolved plastic strain, $\lambda_{\mathrm{int}ra}$ and $d_{\mathrm{int}ra}$ are the average intragranular oxide particle spacing and size, respectively, $n_\perp$ is the rate of dislocations arrival at a nanoparticle. It can be found from eq 4 that the increase rate of stored dislocation density is proportional to the volume fraction of oxide nanoparticles and inversely proportional to their size. Given a certain amount of intragranular oxide particle, the increase rate of stored dislocation density will be significant when the size of oxide particle is reduced to below 5 nm. The calculated value is 5.71×10^{16} m^{-2}, about an order of magnitude higher than the value in previous work[12,13], indicating that the high-density intragranular oxide nanoparticles dominates the strain hardening of cWY alloy.

Supplementary References

1. Lian, Y. *et al.* Mechanical properties and thermal shock performance of W-Y_2O_3 composite prepared by high-energy-rate forging. *Phys. Scr.***T170**, 014044 (2017).
2. Xie, Z. M. *et al.* Effect of high temperature swaging and annealing on the mechanical properties and thermal conductivity of W-Y_2O_3. *J. Nucl. Mater.* **464**, 193-199 (2015).
3. Liu, R. *et al.* Nanostructured yttria dispersion-strengthened tungsten synthesized by sol-gel method. *J. AlloysCompd.***657**, 73-80 (2016).
4. Zhang, T. *et al.* Recent Progress of Oxide/Carbide Dispersion Strengthened W-Based Materials. *Acta Metall. Sin.***54**, 831-843 (2018).
5. Tan, X. Y. *et al.* Mechanical properties and microstructural change of W-Y_2O_3 alloy under helium irradiation. *Sci. Rep.***5**, 12755 (2015).
6. Battabyal, M. *et al.* Microstructure and mechanical properties of a W-2wt.%Y_2O_3 composite produced by sintering and hot forging. *J. Nucl. Mater.***442**, S225-S228 (2013).
7. Xie, Z. M. *et al.* Achieving high strength/ductility in bulk W-Zr-Y_2O_3 alloy plate with hybrid microstructure. *Mater. Des.***107**, 144-152 (2016).
8. Gladman, T. Precipitation hardening in metals. *Mater. Sci. Technol.***15**, 30-36(1999).
9. Kelly, P. M. Progress report on recent advances in physical metallurgy. III. The quantitative relationship between microstructure and properties in two-phase alloys. *Int. Metall. Rev.***18**, 31-36 (1973).
10. Cordero, Z. C., Knight, B. E. & Schuh, C. A. Six decades of the Hall-Petch effect - a survey of grain-size strengthening studies on pure metals. *Int. Mater. Rev.***61**, 495-512 (2016).
11. Teixeira, J. D., Bourgeois, L., Sinclair, C. W. & Hutchinson, C. R. The effect of shear-resistant, plate-shaped precipitates on the work hardening of Al alloys: Towards a prediction of the strength-elongation correlation. *Acta Mater.***57**, 6075-6089 (2009).
12. Huang, L. *et al.* In situ oxide dispersion strengthened tungsten alloys with high compressive strength and high strain-to-failure. *Acta Mater.***122**, 19-31(2017).
13. Liu, G. *et al.* Nanostructured high-strength molybdenum alloys with unprecedented tensile ductility. *Nat. Mater.***12**, 344-350(2013).
